# Identification of bottlenecks in antibody expression using targeted gene integration

**DOI:** 10.1186/1753-6561-9-S9-P7

**Published:** 2015-12-14

**Authors:** Patrick Mayrhofer, Alexander Mader, Bernhard Kratzer, David Reinhart, Willibald Steinfellner, Wolfgang Sommeregger, Renate Kunert

**Affiliations:** 1 Vienna Institute of BioTechnology (VIBT), Department of Biotechnology, University of Natural Resources and Life Sciences, Vienna, 1190, Austria; 2 Institute of Immunology, Medical University Vienna, Vienna, 1090, Austria

## Introduction

Antibody engineering allows proper design of improved properties in antibody products such as a high binding affinity, stable biophysical properties and low immunogenicity. Besides these obvious features improved by elegant design the primary amino acid sequence also has tremendous effects on the performance of the expression host itself influencing cell culture parameters including specific productivities and growth rates therefore having major impact on the finally obtained antibody expression titers [1]. By understanding the underlying mechanisms how certain primary sequence combinations influence culture performance in conjunction with biophysical features of the antibody protein, we might rationally improve the production process of these expensive but essential biotherapeutic products.

**Figure 1:**
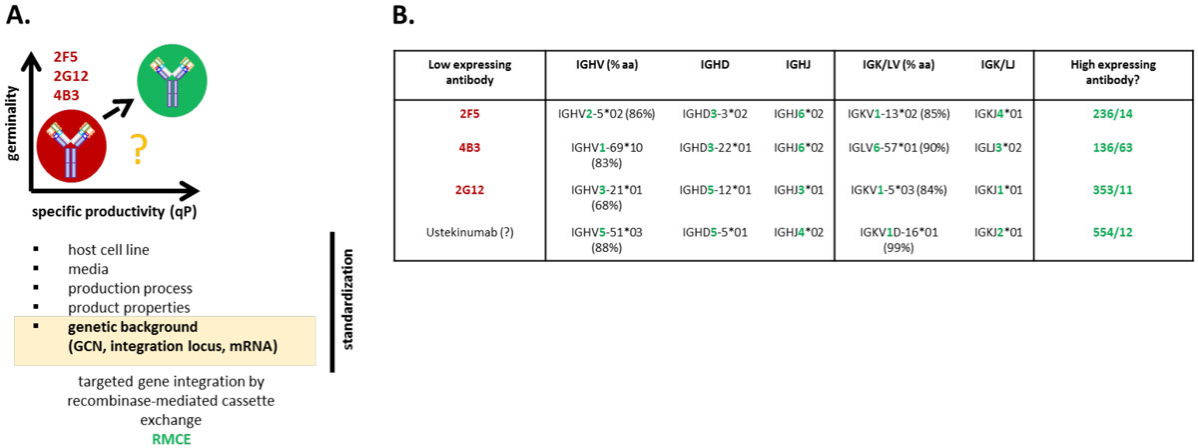
**Observed correlation of specific productivity and sequence identity to the closest human germline sequence (germinality) (**A**)**. Antibody expression levels are influenced by several factors. Recombinase-mediated cassette exchange was applied to control for gene copy number and mRNA transcript level. Low expressing antibody variants (2F5, 4B3 and 2G12) were compared to its closest human germline sequence together with a therapeutic antibody (Ustekinumab) with low degree of germinality (**B**). Respective germline antibody variants were constructed by combination of germline V-, D- and J-segments.

Many different antibody variants were expressed in our group in different projects based on various isotypes. For several antibody variable regions we could observe a direct correlation of advantageous cell performance and a high degree of similarity to the closest human germline sequence of the respective mature antibody leading to higher specific productivities. However, the fundamental principles of these consistent observations and the correlation of cell behavior and biophysical product properties remain quite elusive. Therefore, in this project we aim to set the proper foundations to investigate the generalization of a concept to improve cell performance and antibody expression based on the primary sequence at a high germinality degree (Figure 1). To have proper control of transgene integration locus, gene copy numbers and mRNA transcript level a suitable host for recombinase-mediated cassette exchange (RMCE) was developed to compare different antibody variants under isogenic conditions.

## Materials and methods

All cell lines (CHO-K1, DUKX-B11) were grown under serum- and protein-free conditions in chemically defined media in suspension using shaker flasks. Transfections were performed with the cationic 25kDa linear polymer polyethylenimine (PEI). Stable subclones were either generated by limited dilution subcloning or fluorescence-activated cell sorting (FACS).Germline antibody sequences were identified by the IgBlast program. Variable antibody sequences were synthesized and cloned into vectors containing leader and constant antibody regions.

For recombinase-mediated cassette exchange a CMV (for DUKX-B11) or CAGGS (for CHO-K1) promoter was placed 5' upstream of the first flippase recognition target site to establish a promoter trap in the parental RMCE-cassette. A selection/reporter marker was placed in-between two heterospecific FRT sites harboring a gfp/thymidine-kinase/neomycin-phosphotransferase fusion protein. Antibody producing cells were developed after recombination with the reporter marker by negative selection with ganciclovir. Antibody product was purified by protein A chromatography.

## Results

The observed expression levels in cell cultures are influenced by many different parameters that should be controlled for a valid comparison of different antibody variable sequences. Besides different host cell lines and media formulations our group also has standardized fermentation process protocols and biophysical analytical tools for in-depth investigations of possible correlation between cell culture performance, product quality attributes and expression levels. Recently, we also engineered a CHO DUKX-B11 host cell line suitable for recombinase-mediated cassette exchange (RMCE) [2] to control the integration locus by targeted gene integration leading to equal gene copy numbers (GCN) and mRNA levels between different subclones or even between different antibody variants [3]. Although we could demonstrate a single RMCE-integration locus, facilitating the generation of isogenic cell lines, the overall productivity remains relatively low. Therefore a second generation RMCE host cell line was designed based on CHO-K1 using the CAGGS promoter as a promoter-trap. To analyze a panel of low expressing antibodies in comparison to germline antibodies the primary amino acid sequence was aligned to the closest human germline sequence. The low expressing anti-HIV antibodies 2F5, 2G12 and 4B3, as well as the therapeutic anti-IL12/23 antibody Ustekinumab show quite low levels of homology to its closest human germlines (germinality) (Figure 1B). Based on the aligned germline sequences novel germline antibodies were constructed by combining the respective human germline sequences (236/14, 353/11, 136/63 and 554/12). This panel of 8 antibodies, spanning anti-HIV or therapeutic antibodies as well as kappa and lambda light chain sequences, will be expressed under isogenic and standardized conditions to investigate critical cell culture parameters such as specific productivity (qP) and growth rates (µ), product titer, metabolism, gene copy number, mRNA transcript level or intracellular product content. Additionally, enough material will be expressed based on the improved RMCE host cell line for proper investigation of biophysical properties of the antibody molecules such as thermal and chemical stability, aggregation and oligomer formation propensity or glycosylation profiles.

## Conclusions

An improved RMCE host cell line and an antibody panel consisting of versatile variable regions including mature antibodies and respective germline antibody sequences were constructed for in-depth analysis of cell culture parameters and biophysical properties evoked by different degrees of germinality. By understanding the underlying principles it should be possible to improve antibody cell culture processes and biophysical features by means of proper antibody engineering to optimize the primary antibody sequence.
